# Renal Denervation Attenuates Neuroinflammation in the Brain by Regulating Gut-Brain Axis in Rats With Myocardial Infarction

**DOI:** 10.3389/fcvm.2021.650140

**Published:** 2021-04-26

**Authors:** Jun-Yu Huo, Wan-Ying Jiang, Yi-Ting Lyu, Lin Zhu, Hui-Hui Liu, Yuan-Yuan Chen, Meng Chen, Jie Geng, Zhi-Xin Jiang, Qi-Jun Shan

**Affiliations:** Department of Cardiology, The First Affiliated Hospital of Nanjing Medical University, Nanjing, China

**Keywords:** renal denervation, myocardial infarction, neuroinflammation, intestinal injury, gut microbiota

## Abstract

**Aims:** The development of neuroinflammation deteriorates the prognosis of myocardial infarction (MI). We aimed to investigate the effect of renal denervation (RDN) on post-MI neuroinflammation in rats and the related mechanisms.

**Methods and Results:** Male adult Sprague-Dawley rats were subjected to sham or ligation of the left anterior descending coronary artery to induce MI. One week later, the MI rats received a sham or RDN procedure. Their cardiac functions were analyzed by echocardiography, and their intestinal structures, permeability, and inflammatory cytokines were tested. The intestinal microbiota were characterized by 16S rDNA sequencing. The degrees of neuroinflammation in the brains of rats were analyzed for microglia activation, inflammatory cytokines, and inflammation-related signal pathways. In comparison with the Control rats, the MI rats exhibited impaired cardiac functions, intestinal injury, increased intestinal barrier permeability, and microbial dysbiosis, accompanied by increased microglia activation and pro-inflammatory cytokine levels in the brain. A RDN procedure dramatically decreased the levels of renal and intestinal sympathetic nerve activity, improved cardiac functions, and mitigated the MI-related intestinal injury and neuroinflammation in the brain of MI rats. Interestingly, the RDN procedure mitigated the MI-increased intestinal barrier permeability and pro-inflammatory cytokines and plasma LPS as well as ameliorated the gut microbial dysbiosis in MI rats. The protective effect of RDN was not significantly affected by treatment with intestinal alkaline phosphatase but significantly reduced by L-phenylalanine treatment in MI rats.

**Conclusions:** RDN attenuated the neuroinflammation in the brain of MI rats, associated with mitigating the MI-related intestinal injury.

## Introduction

Myocardial infarction (MI) is one of the most dangerous diseases that threaten public health. Although emergency revascularization of the coronary artery has been widely applied, long-term complications, particularly for long-term cognitive impairment post MI, remain a big challenge (1). Currently, there is still no specific treatment for improving cognitive impairment in MI patients. A recent study has pointed that neuroinflammation is crucial for the development of cognitive deterioration (2). Neuroinflammation can occur in the early stage of functional impairment in the central nervous system (CNS) (3) and have been observed in both MI mice and coronary heart disease (CHD) patients (4). Thus, scientific research approaches to improvement of neuroinflammation following MI recovery will be of high significance in management of patients with cognitive impairment after MI.

Recent studies have revealed that intestinal microbiota and the gut-brain axis are important for regulating the development of CNS disease (5). Physiologically, intestinal health and its related microbial balance can promote the production of neuromodulators to regulate the CNS development, immune responses, and the blood-brain barrier (BBB) function (6). However, in pathological state, the imbalance of gut microbial compositions and the damages of gut barrier can produce and allow more harmful products into the systemic circulation (7). This process can further impair the BBB function and activate microglia, leading to neuroinflammation (8). Interestingly, recent studies have also reported intestinal dysfunction and microbiota imbalance in cardiovascular diseases (9). MI may particularly trigger gut barrier failure and intestinal microbial transportation (10). Hence, intestinal microbiota may be a vital linker of MI and CNS diseases, which may serve as a potential therapeutic target for the improvement of MI-related neuroinflammation.

With regard to effectively improving the intestinal imbalance, it has become evident that the gut state and function are closely related to sympathetic activity (11). In many digestive diseases, the increased sympathetic activity can impair the intestinal barrier function, deteriorate intestinal inflammation (12), and change the composition of intestinal microbiota (13). Furthermore, high sympathetic activity can also promote intestinal pathological changes and intestinal microbial imbalance in hypertensive rats (14). In addition, treatment with a regional sympathetic blocker does effectively alleviate gut injury and microbiota transportation (15). Therefore, a reduction in long-term sympathetic activity may be a potential strategy to improve intestinal abnormalities after MI.

Renal denervation (RDN) is a unique procedure to remove renal nerves for the regulation of sympathetic activity and has been shown to ameliorate hypertension (16). Besides decreasing blood pressure, RDN may extend to treatment of cardiac disease. Researchers have shown that RDN can restore the activity of neuronal nitric oxide synthase in the paraventricular nucleus (17) and improve the central sympathetic nerve remodeling (18), which may eventually attenuate the systemic sympathoexcitation. Theoretically, based on the regulation of systemic sympathetic activity, RDN may improve intestinal abnormalities and neuroinflammation after MI. However, there is no information on the therapeutic effect of RDN on post-MI intestinal abnormalities and how RDN regulates the related CNS neuroinflammation after MI.

Thus, the present study aimed to first explore the potential relationship between intestinal abnormalities and CNS neuroinflammation and then examine the protective effect of RDN on neuroinflammation and potential mechanisms in MI rats.

## Materials and Methods

### Animal Study Design and Sample Collection

**Figure 1:**
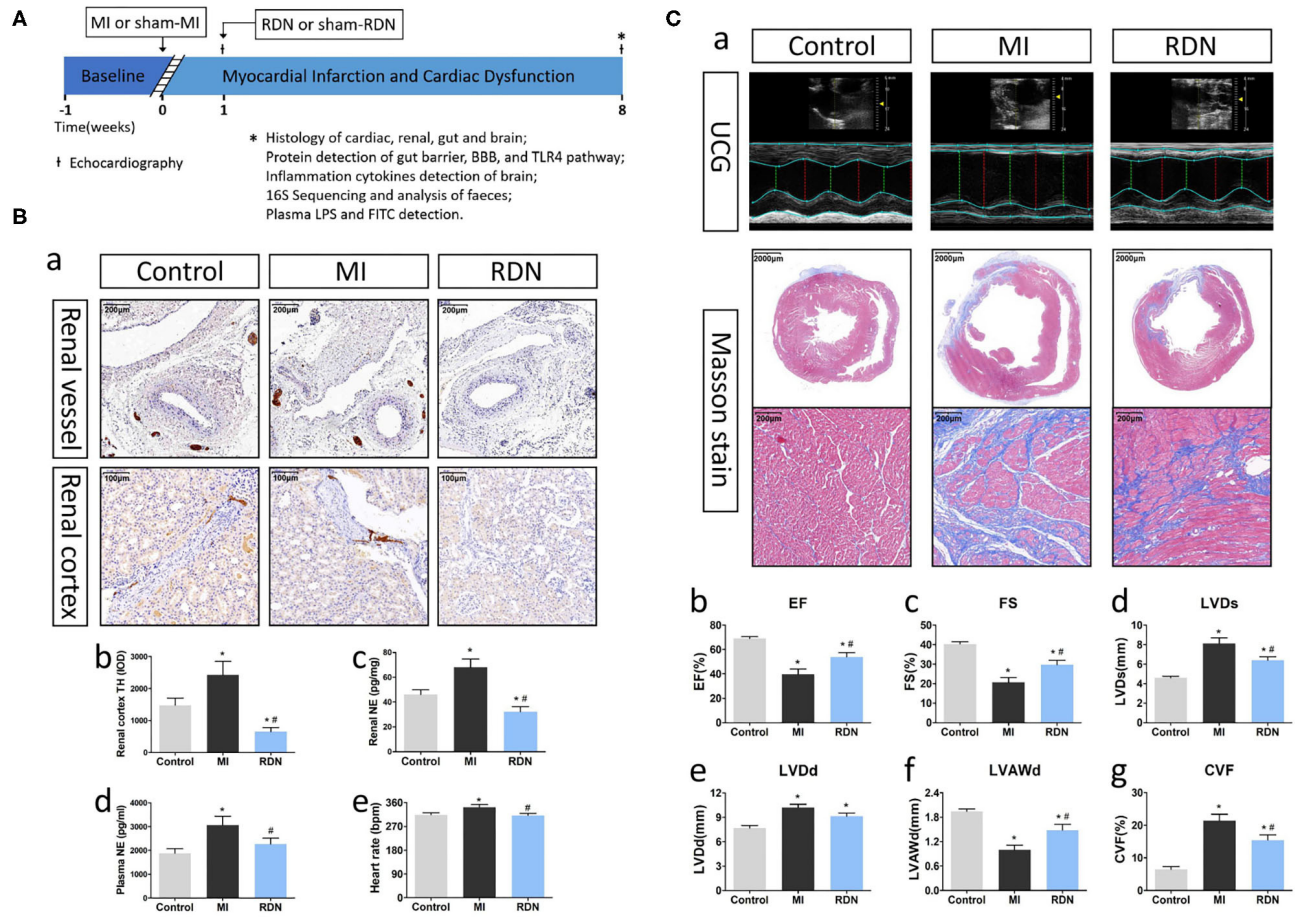
RDN removes renal sympathetic nerve and ameliorates cardiac function in MI rats. **(A)** A schematic diagram illustrates the experimental protocol. **(B)** RDN significantly decreases renal sympathetic nerve activity. (a) Representative images of anti-TH immunohistochemical staining in the renal vessel (magnification, 100×) and renal cortex (magnification, 200×) of rats. (b) Quantitative analysis of TH-positive regions in the renal cortex. (c) ELISA analysis of renal NE levels. (d) ELISA analysis of plasma NE levels. (e) Heart rate of rats from each group. **(C)** RDN ameliorates the MI-induced myocardiac dysfunction. (a). Echocardiography and Masson's trichrome staining (magnification, 10× and 200×) were performed to evaluate myocardial fibrosis and cardiac function in rats. (b) EF; (c) FS; (d) LVDs; (e) LVDd; (f) LVAWd; (g) CVF. *P < 0.05 vs. the Control group; ^#^*P* < 0.05 vs. the MI group. EF, ejection fraction; FS, fractional shortening; LVDs, left ventricular end systolic diameter; LVDd, left ventricular end diastolic diameter; LVAWd, left ventricular anterior wall diastolic thickness; CVF, collagen volume fraction.

All experimental protocols were approved by the Ethics Committee of Nanjing Medical University and conducted in accord with the European Convention for the Protection of Vertebrate Animals used for Experimental and other Scientific Purposes. Male Sprague-Dawley (SD) rats (6 weeks old) were obtained and caged in a specific pathogen-free room with free access to standard chow and water. After 1-week-acclimation, the rats were randomized and subjected to a sham surgery (*n* = 7, Control) or MI surgery (*n* = 23). At 1 week post-MI, 20 surviving rats randomly received a RDN (RDN group, *n* = 10) or sham procedure (MI group, *n* = 10). The Control rats also received a sham procedure of RDN. The myocardiac function and heart rate of surviving rats were tested by echocardiography immediately before and 7 weeks after the RDN/sham-RDN procedure using a Vevo 2100 system (VisualSonics, Toronto, Ontario, Canada). Then, after 4-h fasting, the rats were tested for intestinal permeability *in vivo*. Subsequently, their blood samples were collected. Then, the rats were euthanized and their fecal samples (from the rectum in a sterile environment), intestine (the similar regions), brain, heart, kidneys, and renal vessels were harvested (Figure 1A).

Additional 60 SD rats also induced MI to verify the relationship between RDN's protection on intestinal abnormalities and its prevention of neuroinflammation using intestinal barrier protector intestinal alkaline phosphatase (IAP) (19) and intestinal homeostasis inhibitor L-phenylalanine (L-phe) (20). Briefly, 1 week after MI, all the rats were randomly divided into six groups: MI group (sham RDN), RDN group (RDN), IAP group (sham RDN+IAP), RDN+IAP group (RDN+IAP), L-phe group (sham RDN+L-phe), and RDN+L-phe group (RDN+L-phe). The rats in the IAP group were given drinking water containing 160 units/ml of IAP (21). The rats in the L-phe group were treated with 100 mM L-phe in saline (1.6 ml) by gavage twice per day and other groups of rats received the same quantity of saline (22). Eight weeks after MI, the intestinal permeability was tested and the samples were collected as described above.

### MI Model

The MI model was established by ligating the left anterior descending coronary artery, as reported previously (4). Briefly, rats were injected intraperitoneally (i.p.) with 2% sodium pentobarbital (50 mg/kg), endotracheally intubated and mechanically ventilated. Their left thoracotomy surgery was performed at the fourth intercostal space, and their left anterior descending coronary artery was ligated with a 7–0 silk suture at ~1–2 mm from the branching point, followed by suturing their thoracic cavity and skin. The Control rats received a thoracotomy surgery without ligation.

### RDN Procedure

A RDN procedure was performed, as described previously (23). Briefly, after being anesthetized, rat kidneys and peri-renal adipose tissues were exposed and their all-visible nerves were severed, followed by painting the renal vessels with 20% of phenol in alcohol. The rats in the sham group received the procedure without nerve destruction.

### Intestinal Permeability *in vivo*

Intestinal permeability in individual rats was evaluated using FITC-labeled dextran (24). Briefly, after a 4-h fasting, the rats were gavaged with 50 mg/100 g body weight FITC-labeled dextran 4 kDa (Sigma-Aldrich, St. Louis, USA). Three hours later, their blood samples were collected and their fluorescent signals were quantified in triplicate in a fluorescence spectrophotometer (excitation, 490 nm; emission, 520 nm), according to a standard curve established using different concentrations of FITC-dextran.

### Histological Analysis

Their heart and intestinal tissue sections were routine-stained with Masson's trichrome and hematoxylin–eosin (HE), respectively. For the analysis of Masson staining, five fields from each sample were randomly selected, and the collagen volume fraction (CVF) was assessed using Image-Pro Plus 6.0 software (National Institutes of Health, NIH).

### Immunohistochemistry

The efficacy of RDN in individual rats was evaluated for tyrosine hydroxylase (TH) expression in the renal vessel and renal cortex tissue sections by immunohistochemistry using anti-TH antibody (Servicebio, Wuhan, China). The intensity of anti-TH staining was determined by measuring their integrated optical density (IOD). The contents of microglia in the cerebral cortex tissues were determined by immunohistochemistry using anti-ionized calcium binding adaptor molecule-1 (Iba-1) antibody (Servicebio).

### Immunofluorescence

The distribution and levels of tight junction proteins Occludin and ZO-1 as well as TH expression in the intestinal tissues were determined by immunofluorescence. Briefly, the intestinal cryostat sections (5 μm) were subjected to antigen retrieval in citrate buffer and blocked with normal goat sera. The sections were probed with anti-Occludin, anti-ZO-1 (Abcam, Cambridge, UK), or anti-TH (Cell Signaling Technology, Beverly, USA) and reacted with Alexa Fluor-conjugated secondary antibodies, followed by nuclear staining with 4',6-diamidino-2-phenylindole (DAPI; Servicebio). CD68 and mitochondrial translocator protein (TSPO) are important markers when microglia were activated and neuroinflammation occurred (4). In order to detect the distribution and levels of CD68 and TSPO, immunofluorescence was similarly performed in the cerebral cortex sections using anti-CD68 and anti-TSPO (Cell Signaling Technology) as well as appropriate Alexa Fluor-labeled secondary antibodies. Their fluorescent signals were examined using a confocal laser microscope (Nikon, Tokyo, Japan).

### Measurement of Gut Microbiota

Microbial composition in individual fecal samples was analyzed by 16S rDNA gene sequencing. Briefly, fecal DNA from individual fecal samples was extracted using a PowerFecal DNA Isolation Kit (Qiagen-Mobio, German), according to the manufacturer's instructions. The V3/4 region of the 16S rDNA was amplified by PCR using the primers 341F (CCTACGGGNGGCWGCAG) and 785R (GACTACHVGGGTATCTAATCC). The PCR products were sequenced in an Illumina MiSeq Sequencer (SeqMatic, USA). The raw data were filtered, processed, and analyzed, according to the QIIME quality controlled process (Version1.7.0). The same operational taxonomic units consisted of sequences with ≥97% similarity. The taxonomic annotation was made using RDP classifier and GreenGene Database. The complexity of species diversity in a sample was analyzed using three major α diversity parameters, including Chao1 richness, Simpson index, and Shannon index. The microbial difference among three groups was analyzed by principal component analysis (PCA) using QIIME. The data files can be viewed at NCBI SRA accession PRJNA704634.

### Western Blotting

Occludin and ZO-1 are vital tight junction proteins to maintain barrier function (25). Toll-like receptor 4 (TLR4) and its downstream myeloid differentiation factor 88 (MyD88) form an important pathway to promote the activation of microglia (26). The relative levels of Occludin and ZO-1 expression in the intestine and brain and TLR4 and MyD88 expression in the brain to the β-actin were quantified by Western blotting. Briefly, each type of tissue samples were homogenized in lysis buffer (Servicebio) and centrifuged. After determining protein concentrations using the bicinchoninic acid method, the lysates (30 μg/lane) were separated by sodium dodecyl sulfate–polyacrylamide gel electrophoresis on 10% gels and transferred onto polyvinylidene fluoride membranes. The membranes were blocked with 5% non-fat dry milk in phosphate-buffered saline (PBS) containing Tween 20 and probed with anti-Occludin, anti-ZO-1, anti-TLR4, and anti-MyD88 (Cell Signal Technology) at 4°C overnight. After being washed, the bound antibodies were detected with appropriate horseradish peroxidase (HRP)-conjugated secondary antibody (Servicebio) and visualized with enhanced chemiluminescence.

### Enzyme-Linked Immunosorbent Assay (ELISA)

The collected blood samples were centrifuged to prepare plasma samples. Similarly, fresh kidney, intestinal, and brain tissues were homogenized and centrifuged, respectively. The levels of norepinephrine (NE) in individual renal samples and plasma were determined using the specific kit (Cusabio). The levels of TNF-α, IL-6, and IL-1β in the brain and intestinal samples were analyzed by ELISA using specific kits (Servicebio). The levels of lipopolysaccharide (LPS) in the blood sample were analyzed by ELISA using a specific kit (Cusabio).

### Statistical Analysis

Quantitative data are expressed as the mean ± SEM. The difference among groups was analyzed by one-way ANOVA followed by the Newman-Keuls test using SPSS 16.0 software (SPSS, Chicago, USA). Alpha and beta diversities of 16S rDNA sequencing data were analyzed by two-way repeated measured (ANOVA) and analysis of similarity (ANOSIM) using the QIIME software package. *P* < 0.05 was considered statistically significant.

## Results

### RDN Removes Renal Sympathetic Nerve and Ameliorates Cardiac Function in Mi Rats

At 8 weeks post-MI, there were 7, 6, and 8 surviving rats in the Control, MI, and RDN groups, respectively. Following the RDN procedure, the TH-positive regions in renal vessels were significantly reduced, and there was a significant reduction in the levels of anti-TH staining and NE in the renal tissues of rats, as well as in the level of plasma NE and heart rate (Figures 1Ba–e), demonstrating effective inactivation of sympathetic nerves in the kidneys of rats.

Eight weeks after MI, Masson's staining exhibited that the rats in the MI group displayed dilated left ventricular, a weakened anterior wall in the left ventricular, and obvious fibrous scar formation in the heart (Figures 1Ca,g). Similarly, echocardiography showed that MI increased left ventricular end-systolic diameter (LVDs) and left ventricular end-diastolic diameter (LVDd), decreased left ventricular anterior wall diastolic thickness (LVAWd), as well as reduced ejection fraction (EF) and fractional shortening (FS) of the left ventricular (Figures 1Cb–e). These pathological and functional changes were hallmarks of MI. More importantly, RDN significantly mitigated the MI-related abnormal cardiac structural and functional changes at 8 weeks post MI (Figure 1C). Moreover, echocardiography also revealed that there was no significant difference in the cardiac functional measures between the MI and RDN groups before RDN procedure (Supplementary Figure 1). Hence, RDN effectively removed kidney sympathetic nerves and ameliorated cardiac function in MI rats.

### RDN Mitigates the MI-Related Intestinal Injury

**Figure 2:**
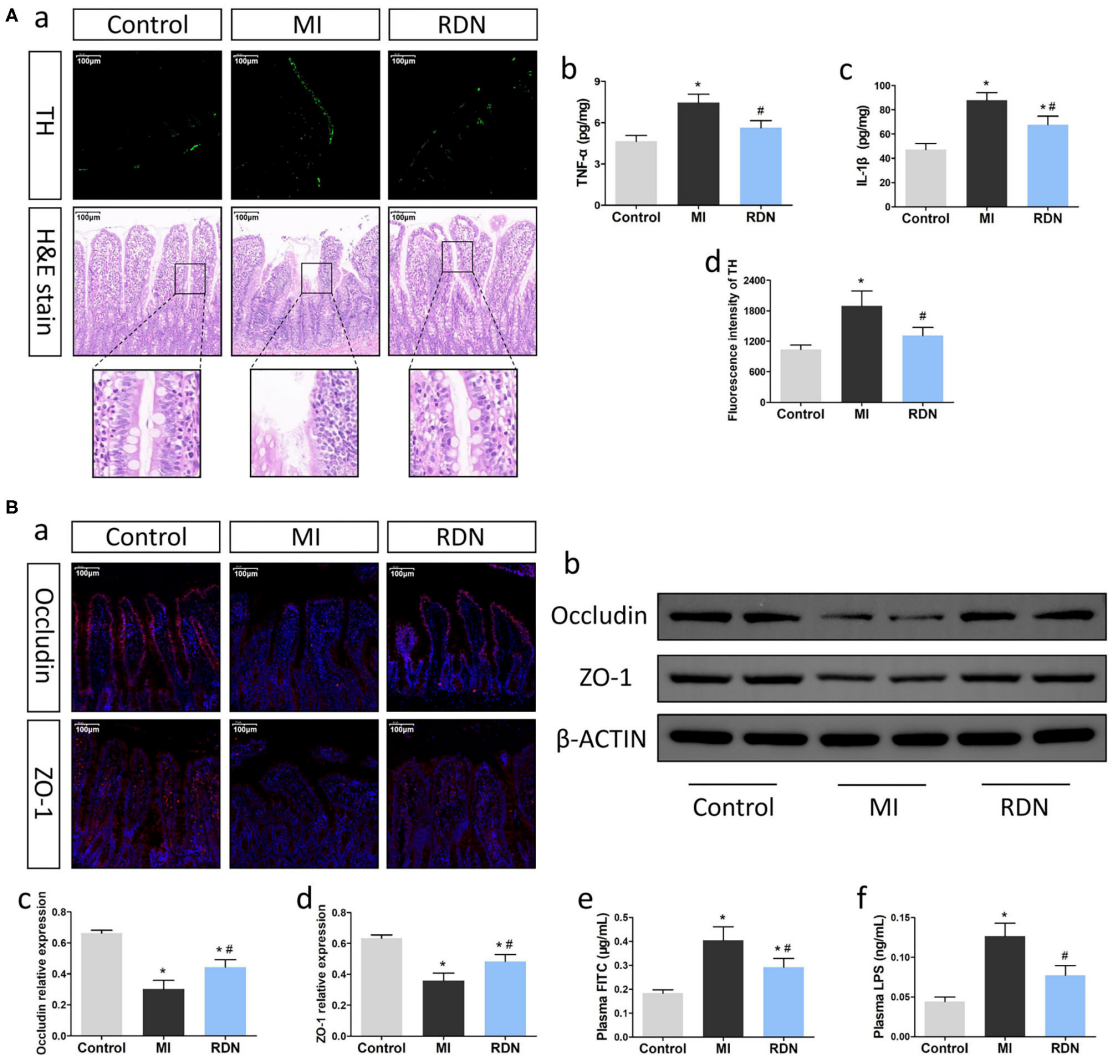
RDN effectively improves the intestinal injury in MI rats. **(A)** RDN reduces the intestinal sympathetic nerve activity and decreases the levels of intestinal pro-inflammatory cytokines to preserve the intestinal barrier integrity in MI rats. (a) Representative images of anti-TH immunofluorescent staining (magnification, 200×) and HE staining (magnification, 200×) in the intestinal tissues of rats. (b,c) ELISA analysis of intestinal TNF-α and IL-1β levels. (d) Quantitative analysis of anti-TH fluorescent signals in the intestine. **(B)** RDN mitigates the MI-increased intestinal permeability in rats. (a) Representative immunofluorescent images of anti-Occludin and anti-ZO-1 staining in the intestine of each group of rats (magnification, 200×). (b) Western blotting analysis of the relative levels of Occludin, ZO-1, and the control β-actin expression in intestinal tissues of rats. (c,d) Quantitative analysis of Occludin and ZO-1 expression. (e) The plasma FITC-dextran concentrations in rats. (f) The levels of plasma LPS in rats. Data are representative images or expressed as the mean ± SEM of each group of rats from three separate experiments. *P < 0.05 vs. the Control group; ^#^*P* < 0.05 vs. the MI group.

MI is usually associated with intestinal injury, impairing intestinal barrier function. To explore whether RDN could modulate the MI-related intestinal injury, we examined intestinal tissues at 8 weeks post MI. Firstly, anti-TH immunofluorescent staining displayed that the fluorescent signals in the intestinal tissues of the RDN group were significantly lower than that in the MI group (Figures 2Aa,d). Secondly, compared with healthy intestinal morphology in the Control group, the MI group exhibited intestinal villi atrophy and damages, as well as reduced numbers of goblet cells accompanied by shed villus epithelium, which were obviously alleviated in the RDN group (Figure 2Aa). Moreover, analysis of intestinal cytokines indicated that the levels of intestinal TNF-α and IL-1β in the RDN group were significantly lower than that in the MI group (Figures 2Ab,c). Thus, RDN mitigated the MI-related intestinal damages.

### RDN Improves Intestinal Barrier Function in Rats

Next, we determined the effect of RDN on intestinal barrier function by measuring the levels of Occludin and ZO-1 expression, two tight junction proteins for intestinal barrier (27). Compared with that in the Control, immunofluorescence indicated significantly decreased anti-Occludin and anti-ZO-1 signals in the MI group, which was partially restored in the RDN group (Figure 2Ba). A similar pattern of Occludin and ZO-1 expression in the intestinal tissues of different groups was detected by Western blot (Figures 2Bb–d). Furthermore, analysis of intestinal permeability using FITC-labeled dextran revealed that the levels of plasma FITC-dextran in the RDN group were significantly lower than that in the MI group (Figure 2Be). LPS, an important metabolite of gut microbiota, can be released into circulation due to the gut barrier leakage (28). With the increase of intestinal permeability, the levels of circulating LPS were markedly elevated in MI rats, compared to the Control rats; RDN effectively mitigated the increased levels of circulating LPS (Figure 2Bf). Such data indicated that RDN improved intestinal barrier function.

### RDN Ameliorates Intestinal Dysbiosis

**Figure 3:**
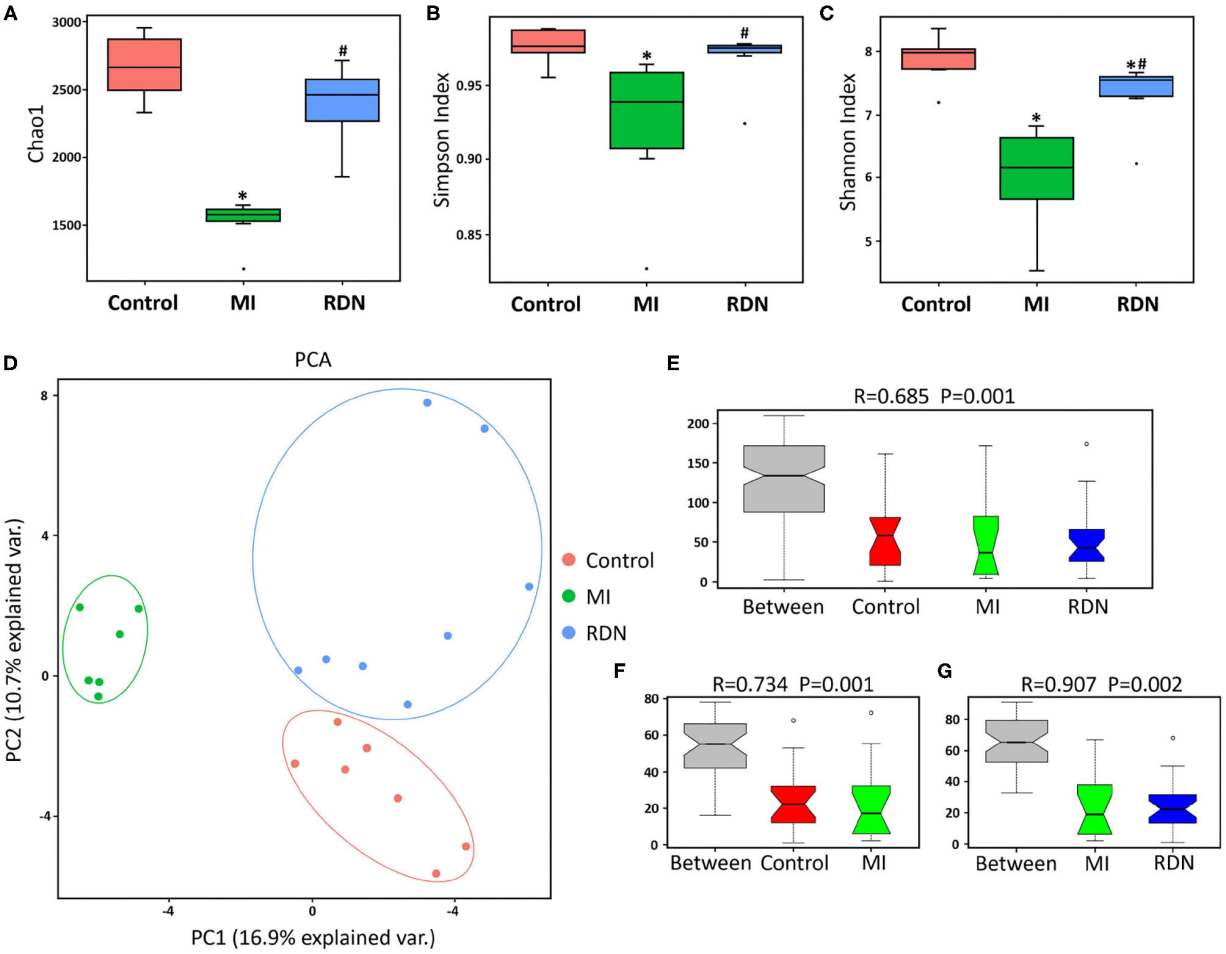
RDN mitigates gut microbiota dysbiosis in MI rats. **(A)** The Chao 1 richness in different groups. **(B)** The Simpson diversity in different groups. **(C)** The Shannon diversity in different groups. **(D)** Principal component analysis of the similarity of microbial communities among these groups. **(E)** ANOSIM analysis of P and R values indicates the difference in microbial community structure between groups. **(F)** ANOSIM analysis of P and R values indicates the difference in microbial community structure between the Control and MI groups. **(G)** ANOSIM analysis of P and R values indicates the difference in microbial community structure between the MI and RDN groups. *P < 0.05 vs. the Control group; ^#^*P* < 0.05 vs. the MI group.

To understand the effect of RDN on intestinal injury, we analyzed gut microbial abundance and composition by 16S rDNA sequencing. Compared with that in the Control, the Chao1 richness, Simpson index, and Shannon index in the MI group significantly decreased, which were mitigated by RDN, indicating that RDN ameliorated the MI-decreased gut microbial richness (Figures 3A–C). Then, we also visualized the phylogenetic similarity between microbial communities by principal component analysis and found that there was a significant variation among these groups of rats (Figures 3D,E). Furthermore, compared to the MI rats, the microbial components in the RDN rats converged better with Control rats (Figures 3D,F,G), indicating that RDN made microbial communities relatively stable after MI. Thus, such data indicated that RDN mitigated the MI-decreased gut microbial diversity.

### RDN Ameliorates Neuroinflammation and Preserve the BBB Function in Rats

**Figure 4:**
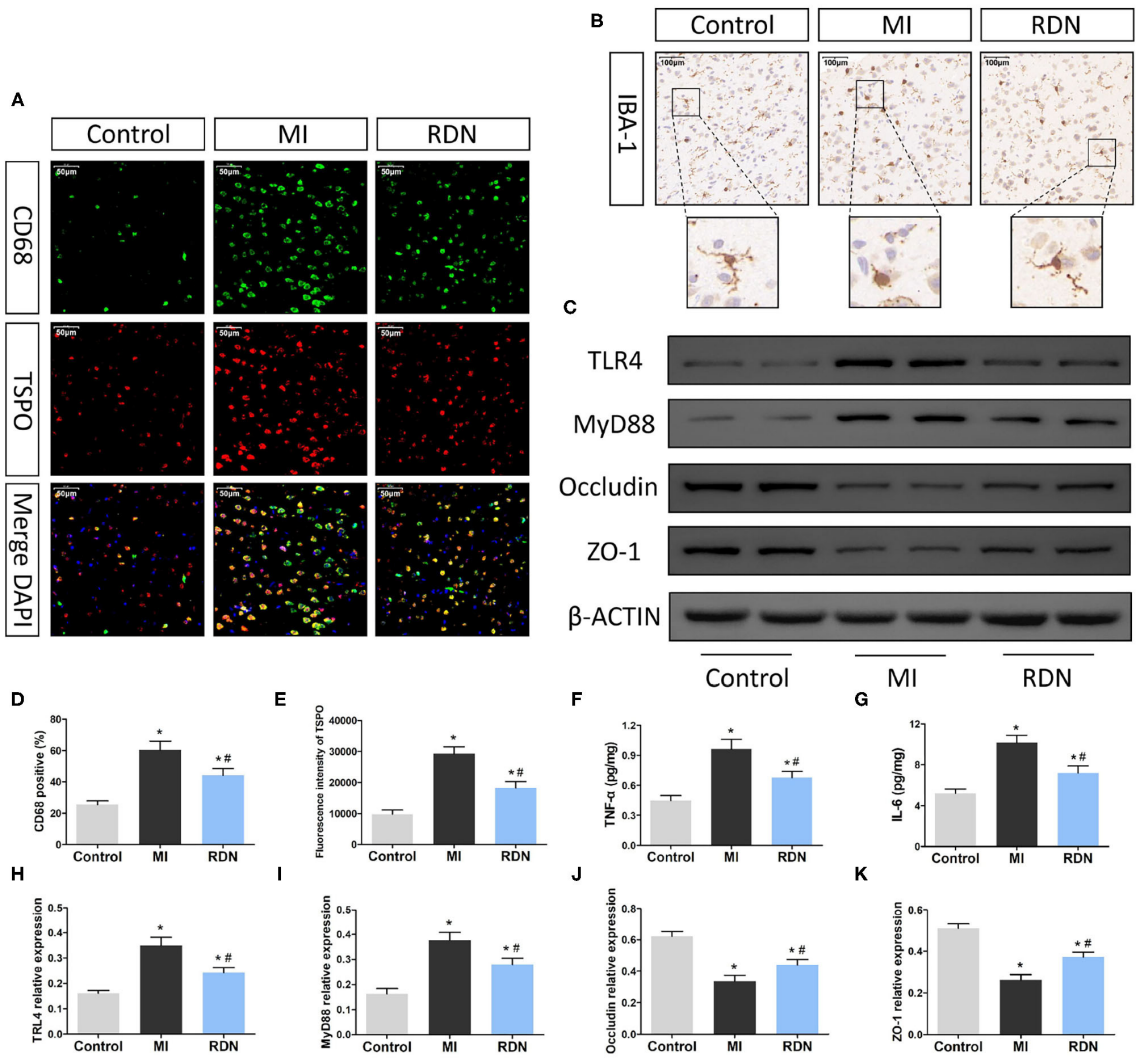
RDN ameliorates the MI-related neuroinflammation in the CNS of rats. **(A)** Immunofluorescent analysis of TSPO and CD68 expression (magnification, 400×) in the brain of rats. **(B)** Immunohistochemsitry analysis of Iba-1 expression (magnification, 200×) in the brain of rats. **(C)** Western blotting analysis of the relative levels of TLR4, MyD88, Occludin, ZO-1, and the control β-actin expression in the brain of rats. **(D)** Quantitative analysis of CD68^+^ microglia in the brain. **(E)** Quantitative analysis of TSPO fluorescent signals in the brain. **(F,G)** ELISA analysis of TNF-α and IL-6 in the brain. **(H–K)** Quantitative analysis of TLR4, MyD88, Occludin, and ZO-1 expression in the brain of rats. Data are representative images or expressed as the mean ± SEM of each group of rats from three separate experiments. *P < 0.05 vs. the Control group; ^#^*P* < 0.05 vs. the MI group. TSPO, translocator protein; DAPI, 4',6-diamidino-2-phenylindole; Iba-1, ionized calcium binding adaptor molecule-1; TLR4, Toll-like receptor 4; MyD88, myeloid differentiation factor 88.

Previous studies have shown that gut abnormalities could, through the gut-brain axis, activate microglia to regulate neuroinflammation in the CNS (26, 29, 30). Given that RDN procedure mitigated the MI-induced intestinal injury we further explored the influence of RDN on post-MI neuroinflammation. CD68 is the marker for microglia activation and TSPO is a transporter protein distributed in the mitochondrial outer membrane, which is highly expressed by activated microglia when inflammatory occurred (4). Immunofluorescence showed that compared with that in the Control group, there were many strong CD68^+^TSPO^+^ microglia in brain cortex of the MI group, but reduced in the RDN group (Figure 4A). Immunohistochemistry indicated that there were many IBA-1^+^ microglia with hypertrophied cytoplasm and retracted processes, in the brain cortex of MI rats, but many IBA-1^+^ microglia still had small cytoplasm and thin tread-like processes in the RDN group, like resting microglia in the Control rats (Figure 4B). Quantitative analysis revealed that the frequency of CD68^+^ microglia and the fluorescent intensity of TSPO staining in the RDN group were significantly lower than that in the MI group (Figures 4D,E). LPS can bind and activate the TLR4 (31) and its downstream inflammatory signaling that is important for neuroinflammation (26). Western blot analysis exhibited that the expression of TLR4 and MyD88 in the brain of the RDN group were significantly lower than that in the MI group (Figures 4C,H,I). Subsequently, we also detected the levels of inflammatory cytokines. Similarly, we observed that TNF-α and IL-6 levels significantly increased in the brain of MI rats, but decreased by RDN treatment (Figures 4F,G).

The BBB is crucial in regulating the central nervous homeostasis and effectively separates the peripheral circulation from the CNS. Endothelial cells in the BBB express tight junction proteins to maintain the function of the BBB. Western blot analysis revealed that the relative levels of Occludin and ZO-1 expression in the brain of the MI group were significantly reduced, relative to that in the Control group, but were partially restored in the RDN group of rats (Figures 4J,K). Collectively, MI induced neuroinflammation and decreased the BBB function in rats, which were significantly mitigated by RDN.

### RDN Attenuates Neuroinflammation Partly by Preserving the Integrity of Intestinal Epithelium in Rats

**Figure 5:**
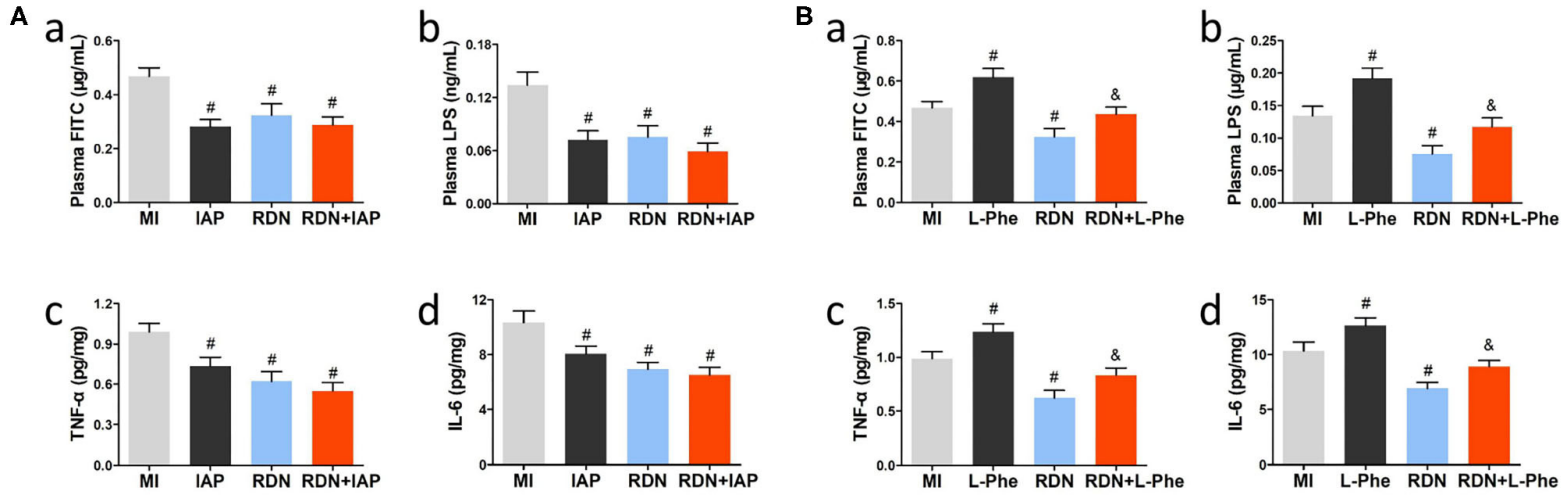
The neuroprotection of RDN is associated with mitigating the MI-related intestinal injury in rats. **(A)** Treatment with IAP, like the RDN procedure, does not enhance the effects of RDN in attenuating intestinal permeability and neuroinflammation in the CNS of MI rats. (a) The plasma FITC-dextran concentrations in rats. (b) The plasma LPS levels in rats. (c,d) ELISA analysis of brain TNF-α and IL-6 in rats. **(B)** Treatment with L-phe mitigates the protective effect of RDN on preserving intestinal barrier integrity and reducing neuroinflammation in the CNS of MI rats. (a) The plasma FITC-dextran concentrations in rats. (b) The plasma LPS levels in rats. (c,d) ELISA analysis of brain TNF-α and IL-6 in rats. *N* = 8, 9, 7, 8, 8, and 7 for the MI, IAP, L-phe, RDN, RDN+IAP, and RDN+L-phe groups, respectively. ^#^*P* < 0.05 vs. the MI group; ^&^*P* < 0.05 vs. the RDN group. IAP, intestinal alkaline phosphatase; L-phe, L-phenylalanine.

To understand the importance of intestinal abnormalities in the MI-related neuroinflammation, we tested whether IAP, a protector of the intestinal epithelium to improve intestinal permeability (32), could modulate the effect of RDN on intestinal permeability and neuroinflammation in MI rats. Following induction of MI and RDN procedure, some rats were randomized and treated with IAP or L-phe. Eight weeks later, we found that treatment with IAP or the RDN significantly decreased the levels of plasm FITC-dextran and LPS, relative to that in the MI rats receiving vehicle treatment (Figures 5Aa,b). Interestingly, combination of IAP and RDN did not further reduce the levels of plasm FITC-dextran and LPS in rats (Figures 5Aa,b). Similarly, treatment with IAP, like the RDN procedure, also significantly decreased the levels of TNF-α and IL-6 in the brain of rats, relative to that in the MI group (Figures 5Ac,d). Combination of IAP treatment and RDN procedure did not further reduce the levels of TNF-α and IL-6 in the brain of rats. In contrast, treatment with L-phe, which could inhibit endogenous IAP activity and injury intestinal homeostasis (20), significantly mitigated the RDN-decreased plasma FITC-dextran and LPS levels in MI rats (Figures 5Ba,b). In addition, treatment with L-phe also significantly attenuated the RDN-reduced TNF-α and IL-6 levels in the brain of rats (Figures 5Bc,d). Together, the RDN procedure preserved the MI-related neuroinflammation in rats partly by preserving the integrity of intestinal epithelial barrier.

## Discussion

### Major Findings

In the present study, we used a rat MI model to evaluate the effect of RDN on the MI-related neuroinflammation in the CNS and its potential mechanisms. We found that RDN not only improved the MI-impaired cardiac functions, but also mitigated intestinal abnormalities as well as neuroinflammation in the CNS of rats. Interestingly, we found that the neuroprotective effect of RDN was partially reduced by L-phe treatment, but was not affected by IAP in MI rats. To the best of our knowledge, this was the first report on RDN mitigating the MI-related intestinal injury and permeability, leading to decreased levels of circulating gut microbial metabolites that activated microglia to promote neuroinflammation.

### MI and Intestinal Abnormalities

Gut microbiota form the largest microbial community in the body. Functionally, gut microbiota can help in absorption of some nutrients, regulate the function of the immune system, and prevent the invasion of pathogens to maintain body health (33). Microbial components are highly influenced by the state of the host. MI induces a severe stress condition to over-activate the neurohumoral system and damage intestinal structure and function, which may feedback cause gut microbial dysbiosis (34). The intestinal injury is attributed to MI-decreased cardiac output and function, leading to digestive tract hypoperfusion, gastrointestinal congestion, ischemia, and edema (35). Furthermore, the aberrant sympathetic nerve activity may impair intestinal function and homeostasis (14). In the present study, we observed obvious pathological alterations and increased inflammation in intestinal villi, accompanied by increased gut barrier permeability in MI rats. These pathogenic changes may also cause microbial dysbiosis (36). In the present study, the 16S rDNA sequencing showed that the microbiota diversities and the composition of intestinal flora were significantly altered after MI, evidenced by the major alpha diversity parameters and principal component analysis. Meanwhile, we also observed increased levels of plasma LPS in MI rats. Consistently, intestinal injury and bacteremia are commonly detected in both MI rats and patients with ST-segment elevation (10). Hence, our data support the notion that MI can cause intestinal injury, alter gut microbiota, and increase the gut barrier permeability.

### Gut-Brain Axis and Neuroinflammation

The gut-brain axis is important for the maintenance of the CNS function. Alternation in gut microbiota is associated with the development of Alzheimer's disease (AD), and drugs targeting intestinal disorders can effectively improve the neuropathology and cognitive impairment in AD mice (37). Similarly, changes in gut microbiota and bacterial metabolites, together with intestinal injury, can promote the entry of bacterial metabolites into the bloodstream to induce neuroinflammation (38). For example, LPS released by gut bacteria can breakdown the BBB and increase its permeability (29). Moreover, LPS can bind to the TLR4 to induce monocyte differentiation into M1-type microglia and activate microglia to express nitric oxide and pro-inflammatory cytokines by activating the TLR4 signaling (8, 30). Evidently, intraperitoneal injections with LPS induced microglia activation and neuroinflammation in CNS in rats (39). Similarly, clinical studies have revealed an increase in the levels of LPS in both the systemic circulation (40) and the brain tissues (41) in AD patients. In the present study, we detected significantly elevated LPS levels and impaired Occludin and ZO-1 expression as well as increased microglia activation and pro-inflammatory TNF-α and IL-6 expression in the brain tissues of MI rats. These data indicated that altered gut microbiota and intestinal injury promoted LPS entry into the bloodstream, which could damage the BBB and activated microglia to induce neuroinflammation in MI rats. Besides, we also treated MI rats with intestinal barrier protector IAP and observed that the intestinal permeability and plasma LPS level were effectively decreased, accompanied by attenuation of neuroinflammation. Therefore, the MI-related intestinal injury, microbiota dysbiosis, and the entry of bacterial metabolites into the bloodstream might be key for MI-induced neuroinflammation, and targeting the microbiota dysbiosis and preserving intestinal integrity may be new strategies to design therapies for the MI-related neuroinflammation.

### The Heart-Gut-Brain Vicious Cycle

During the development of cardiovascular diseases, abnormal activation of the neurohumoral system often leads to the interaction between multiple organs and systems. Thus, besides the influence of MI-related intestinal abnormalities on the brain, cardiovascular disease itself may also affect the state and function of the CNS. On one hand, the heart failure-reduced blood flow velocity could contribute to cerebral hypoperfusion (42) and form microthrombosis in the brain (43), which might aggravate the nerve injury. On the other hand, clinical and basic studies have also shown that the elevated levels of oxidative stress and relative low enzymatic antioxidant defenses caused by MI can promote lipid peroxidation and oxidative damage in the brain (44, 45). Additionally, the deterioration of cardiac function can also result in relative deficiency of neuronutrients such as thiamine (46), together providing the basis for the progress of neuroinflammation after MI.

**Figure 6:**
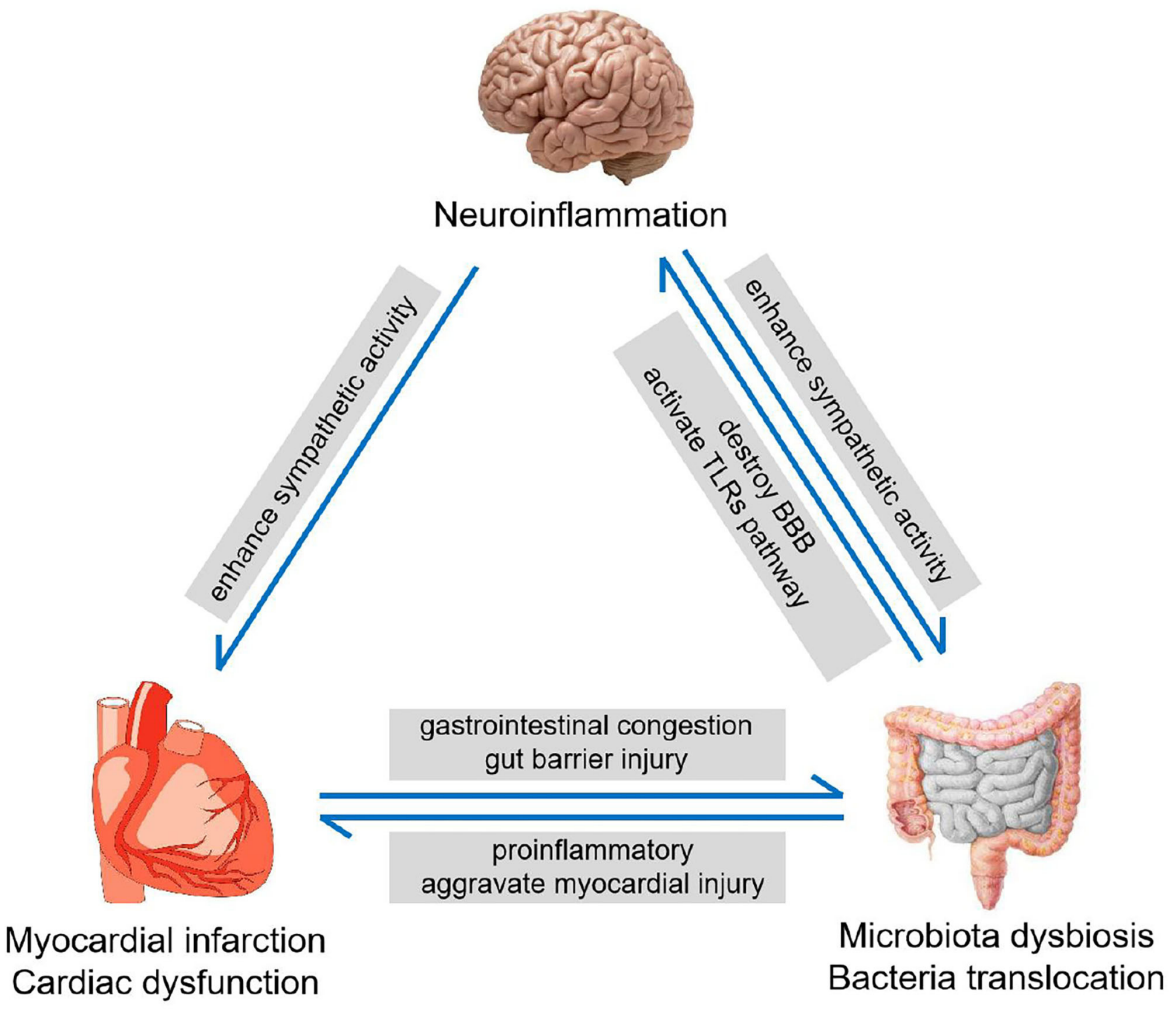
The heart-gut-brain vicious cycle. MI induces the intestinal injury and increases intestinal barrier permeability, leading to microbiota dysbiosis. Microbiota dysbiosis promotes neuroinflammation, which enhances the sympathetic nerve activity and deteriorates myocardial dysfunction.

Likewise, the effects of post-MI intestinal abnormalities may not only be limited to CNS as well. Previous studies have shown that increased bacterial metabolites can affect cardiac function (47) and augment cholesterol accumulation in macrophages (48), which would further aggravate the progression of CHD. Furthermore, the entry of gut bacterial components into the bloodstream can also promote the production of fibrosis and pro-inflammatory factors (10, 49). These indicate that MI can induce intestinal injury, whose feedback affects cardiovascular functions in some pathological conditions. Besides, the elevated neuroinflammation in CNS after MI may also have adverse effects on cardiac and intestinal function. A previous study has shown that the neuroinflammation in the brain can enhance the sympathetic activity in the CNS, which would lead to an increase in the systemic sympathetic activity (50). This would not only deteriorate the cardiac function but also impair intestinal function, to exacerbate gut microbiota dysbiosis. Thus, the interaction between the heart, gut, and brain could form a vicious cycle after MI (Figure 6).

### The Protective Role of RDN After MI

It is widely recognized that the kidney and renal nerves play an important role in neurohumoral activation in cardiovascular disease. Renal sympathetic nerve is the main component of the sympathetic nervous system. The projections of renal afferent nerves to the hypothalamus can stimulate sympathetic outflow and cause systemic sympathetic hyperactivity (51), thus making the heart (52), gut (11), and other organs functionally abnormal. Also, the sympathetic nerve terminating in the nephron could activate β1-adrenergic receptors on the juxtaglomerular apparatus and further stimulate the release of the renin and active renin-angiotensin system (53). The excess angiotensin not only worsens cardiovascular injury but also could affect the intestinal function and promote microbiota abnormalities as well (14, 54). Therefore, RDN may play a unique protective role by alleviating the neurohumoral system after renal nerve removal. Previous studies have suggested that RDN could decrease sympathetic activity and ameliorate heart disease (55). Furthermore, a recent study revealed that RDN could also reduce circulating angiotensin and inhibit the renin-angiotensin system (56), together laying a foundation for RDN's improvement on the abnormality of related organs after MI. In our present study, we verified that RDN not only improved cardiac function but also attenuated gut abnormalities and reduced plasma LPS, as well as improved neuroinflammation in the MI rats. Thus, RDN may be an effective treatment for breaking the heart/gut/brain vicious cycle to improve the prognosis of MI.

### Limitation

Firstly, we acknowledged that our surgical technique to perform RDN is different from clinical catheter ablation. However, the small size of the SD rat renders catheter ablation impractical, and this type of RDN in rats is widely recognized (57). Secondly, we did not record nerve electrical signal for sympathetic activity. However, the electrical signal might not be recorded because the RDN procedure removed all visible nerves, and NE level and TH staining used in the present study may even be recognized as the standardized means in studies of the RDN's effect on sympathetic activity (58, 59). Thirdly, cognitive function was not detected in the present study. However, the association between neuroinflammation and cognitive impairment has been comprehensively studied in basic and clinical studies (3, 60), showing that neuroinflammation could increase the risk of cognitive impairment. Furthermore, further investigations are required to elucidate how the RDN modulates the signal pathways to improve intestinal function and how intestinal injury cause neuroinflammation in the CNS.

## Conclusion

Our data indicated that RDN ameliorated the neuroinflammation in the CNS of MI rats. Mechanistically, RDN improved microbiota dysbiosis and preserve the intestinal barrier integrity, leading to an improvement of neuroinflammation in MI rats. Our findings may provide new insights into the pathogenesis of MI-related neuroinflammation and highlight the importance of the heart-gut-brain axis in regulating body homeostasis.

## Data Availability Statement

The original contributions presented in the study are publicly available. This data can be found here: NCBI repository, accession PRJNA704634.

## Ethics Statement

The animal study was reviewed and approved by Ethics Committee of Nanjing Medical University.

## Author Contributions

All authors listed have made a substantial, direct and intellectual contribution to the work, and approved it for publication.

## Conflict of Interest

The authors declare that the research was conducted in the absence of any commercial or financial relationships that could be construed as a potential conflict of interest.
