# Supplementary material for: Renal Denervation Attenuates Neuroinflammation in the Brain by Regulating Gut-Brain Axis in Rats With Myocardial Infarction
Source: Front Cardiovasc Med. 2021 Apr 26;8:650140. doi: 10.3389/fcvm.2021.650140 (PMC8109795; doi:10.3389/fcvm.2021.650140)

**Supplementary Figure**

**Figure S1. Cardiac functions in rats at 1 week post MI.**

(a). EF; (b). FS; (c). LVDs; (d). LVDd. *P < 0.05 vs. the Control group; # P<0.05 vs. the MI group. EF = ejection fraction; FS = fractional shortening; LVDs = left ventricular end systolic diameter; LVDd = left ventricular end diastolic diameter


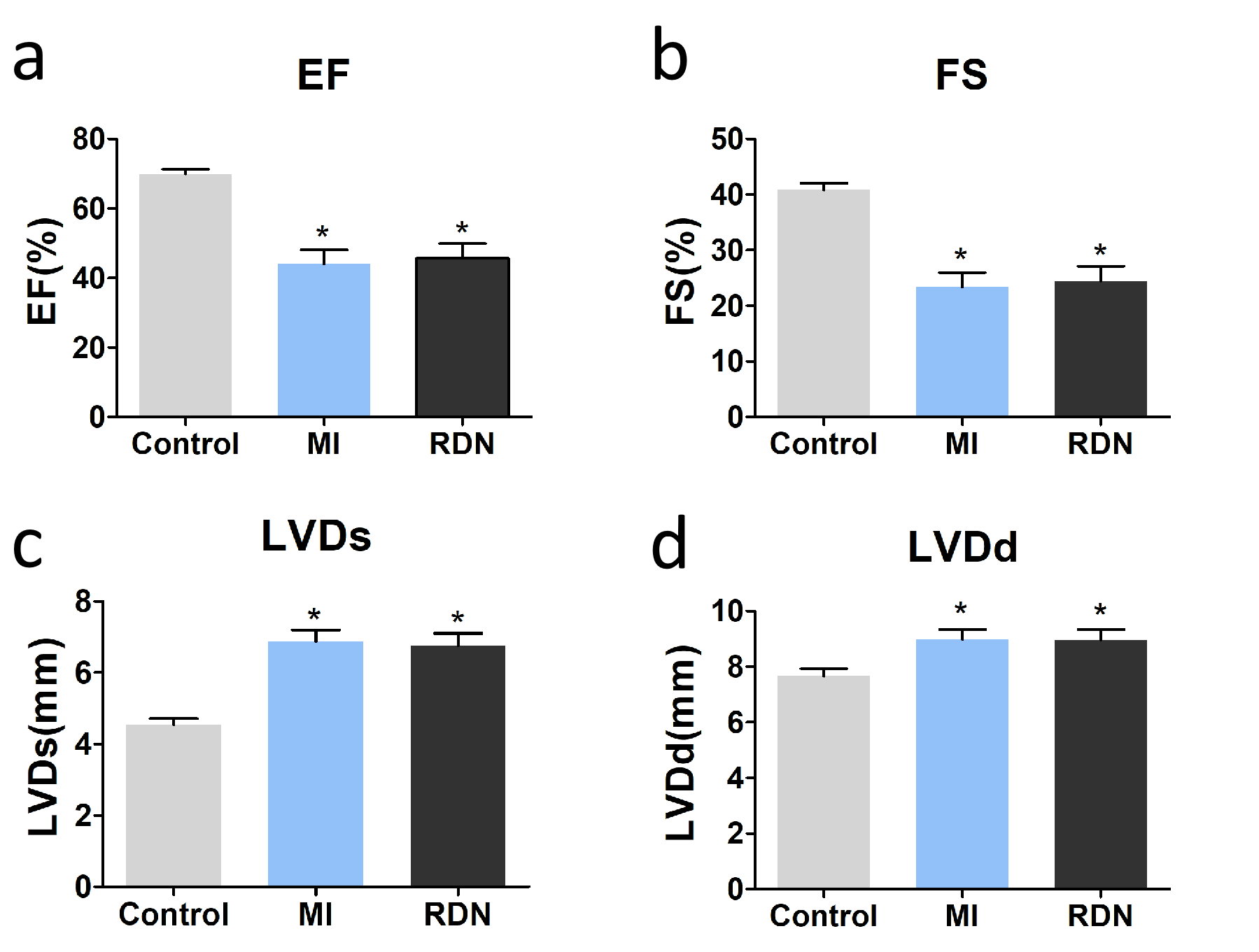

Supplement: Supplementary file 1 [file Data_Sheet_1.doc]
